# A Special, Strict, Fat-Reduced, and Carbohydrate-Modified Diet Leads to Marked Weight Reduction even in Overweight Adolescents with Prader-Willi Syndrome (PWS)

**DOI:** 10.1100/tsw.2009.105

**Published:** 2009-09-14

**Authors:** Walter Bonfig, Kathi Dokoupil, Heinrich Schmidt

**Affiliations:** Dr. Von Hauner Children's, Hospital University of Munich, Germany

**Keywords:** Prader-Willi Syndrome, hyperphagia, low-fat and carbohydrate-modified diet

## Abstract

Hyperphagia is a frequent symptom in patients with Prader-Willi Syndrome (PWS) and results in marked obesity with the risk of metabolic and cardiovascular complications. Previously, we reported that our special diet for PWS patients is effective in the long run, if started early at about 2 years of age. Our objective in this study was to investigate if our special diet is also effective in PWS adolescents who are already overweight. We provided a strict, fat-reduced, and carbohydrate-modified diet, consisting of 10 kcal/cm height, to five adolescents (two female, three male) with PWS. Patients were prospectively followed at our center for 2-6 years. BMI, BMI-SDS, and Weight-for-Height Index were recorded over that period. The special diet was started at a mean age of 16 years (range: 14.1-18.9 years) and initial BMI was 41.3 kg/m (range: 32.4-55.5 kg/m), corresponding to BMI-SDS +3.6 (range: +2.8 to +4.5 SDS). Weight-for-Height Index was 243% (range: 190-339%). After 2 years of the diet, BMI decreased to 33 kg/m (range: 26.7-38 kg/m), as well as BMI-SDS +2.7 (range: 1.7-3.4 SDS) and Weight-for-Height Index to 191% (range: 157-232%); p < 0.01. The special diet was still effective in reducing weight after 4–6 years, with a mean BMI of 30.5 kg/m (range: 24.6–34.5 kg/m) and a mean BMI-SDS of +2.1 (range: 0.7–2.9). We conclude that in a period of 2–6 years, our strict, fat-reduced, and carbohydrate-modified diet, with 10 kcal/cm height, is effective even in adolescents with PWS who are already overweight.

## INTRODUCTION

Prader-Willi Syndrome (PWS) is characterized by muscular hypotonia, feeding difficulties during infancy, retarded motor and mental development, hyperphagia starting at the age of 2–4 years, incomplete pubertal development, and short stature[1]. Weight development in PWS is unique. At birth, weight is slightly reduced. During the first 2 years of life, weight is low or normal due to poor feeding, and this period is followed by rapid weight gain caused by hyperphagia, resulting in a Weight-for-Height Index exceeding the normal range at the age of 10 years in nearly all patients with PWS[2,3]. Obesity, with its metabolic and cardiovascular complications, is the most important life-shortening factor in PWS. Obesity is probably the result of hardly achievable satiety (inborn and of hypothalamic origin) complicated by behavioral abnormalities (inborn and learned). Guidelines for energy intake required for weight loss and weight maintenance for adults, and partially for children with PWS, have been suggested[4,5].

We previously published data on early dietary intervention in PWS, showing that with our special diet, it is possible to prevent uncontrolled weight gain and ameliorate hyperphagia in the long run[6,7]. In this paper, we report on five adolescents with PWS who were already overweight, who underwent the strict, fat-reduced, and carbohydrate-modified diet.

## PATIENTS AND METHODS

Five adolescent patients (two female, three male) with PWS were referred to our outpatient endocrine clinic; they were already obese. Four patients had a paternal deletion of chromosome 15 and one patient had an imprinting mutation on chromosome 15. All five patients were started on a dietary intervention (fat-reduced and carbohydrate-modified regular meals, 10 kcal/cm height). Due to partial hypogonadism, all three males received monthly testosterone substitution, 100–250 mg i.m., and the two females were on a birth control pill. One male also suffered from hypothyroidism and received levothyroxine treatment. All patients were cared for in a foster home. Auxological data were prospectively followed. Height-SDS was calculated by comparing values with published data from Prader et al.[8]. None of the patients had been treated with growth hormone to improve final height. BMI development was recorded and expressed in SDS using British reference data[9] and Weight-for-Height Index was calculated.

Total energy intake was limited to 10 kcal/cm height with three main meals (breakfast, lunch, and dinner) and three snacks. Macronutrients were 25% protein, 20% fat (reduction of one-third of recommended daily fat intake), and 55% modified carbohydrates (significant reduction of mono- and disaccharides). The supply of micronutrients was done according to the recommendation of the German, Austrian, and Swiss References for Nutrition Intake 2000. Strict fat-free or very low-fat diet was recommended (skim milk [<0.3% fat] and dairy products; lean meat, such as turkey, lean beef, or pork; lean fish; whole meal bread; fat-free sweets [biscuits] and snacks [rice crackers]). Low-fat cooking (grill, steam, etc.) was taught, and only small amounts of rapeseed oil for cooking and salad dressing were allowed. A detailed plan for each meal for 7 days/week was created (Table 1). Dietary treatment was taught, supervised, and adapted by the same team (pediatrician and dietician). The staff in the foster home received instruction as well. Regular physical follow-up was done every 6–12 months and patients were asked about hyperphagic phases, as well as food-craving behavior.

Statistical analyses were performed with the nonparametric Mann-Whitney-U Test with the SPSS 10.0 software (SPSS Inc., Chicago). A $p$-value < 0.05 was considered statistically significant.

## RESULTS

The special diet was started at a mean age of 16 years (range: 14.1–18.9 years) and initial BMI was 41.3 kg/m² (range: 32.4–55.5 kg/m²), corresponding to BMI-SDS +3.6 (range: +2.8 to +4.5 SDS). Weight-for-Height Index was 243% (range: 190–339%). Patients were short, with a mean height-SDS of –2.4 (range: –1 to –3.5 SDS), Table 2.

After 1 year, BMI decreased significantly to 34.1 kg/m² (range: 29.7–38.2 kg/m²), $p < 0.01$, as did BMI-SDS from +3.6 to +2.9 SDS (range: +2.4 to +3.6), $p < 0.01$. Weight-for-Height Index dropped from 243 to 197.8% (range: 166–250%), $p < 0.01$.

**TABLE 1**
**Dietary Example for PWS (1600 kcal/day)**

| | | Energy (kcal) | Protein (g) | Fat (g) | Carbohydrate (g) |
|---|---|---|---|---|---|
| Breakfast | | | | | |
| 80 g | Whole grain bread | 170 | 4.7 | 1 | 36 |
| 10 g | Diet margarine | 36 | 0 | 4 | 0 |
| 100 g | Low-fat curd cheese | 75 | 13.5 | 0 | 4 |
| 10 g | Jam | 29 | 0 | 0 | 7 |
| Snack 1 | | | | | |
| 150 g | Fresh fruit | 78 | 0.5 | 1 | 17 |
| 40 g | Pretzel | 136 | 3.8 | 1 | 27 |
| 30g | Low-fat curd cheese | 27 | 4.9 | 0 | 1 |
| Lunch | | | | | |
| 180 g | Steamed potatoes | 126 | 3.6 | 0 | 26 |
| 170 g | Lean pork | 182 | 37.7 | 3 | 0 |
| 150 g | Vegetables | 26 | 1.4 | 0 | 4 |
| 120 g | Salad | 14 | 1.5 | 0 | 1 |
| 10 g | Rapeseed oil | 88 | 0 | 10 | 0 |
| Snack 2 | | | | | |
| 150 g | Yogurt | 57 | 6.4 | 0 | 6 |
| 150 g | Fresh fruit | 78 | 0.5 | 1 | 17 |
| Snack 3 | | | | | |
| 40 g | Russian bread (meringue) | 152 | 3.5 | 0 | 33 |
| Dinner | | | | | |
| 80 g | Whole grain bread | 170 | 4.7 | 1 | 36 |
| 10 g | Diet margarine | 36 | 0.2 | 4 | 0 |
| 90 g | Ham | 112 | 18.8 | 4 | 0 |
| 30 g | Gherkin/pickles | 4 | 0.1 | 0 | 0 |
| 100g | Fresh tomato | 17 | 0.9 | 0 | 3 |
| Total | | 1613 | 106.7 | 30 | 218 |

Also after 2 years, BMI remained significantly lower at 33 kg/m² (range: 26.7–38 kg/m²), as well as BMI-SDS +2.7 (range: 1.7–3.4 SDS) and Weight-for-Height Index 191% (range: 157–232%), all $p < 0.01$.

The special diet was still effective in reducing weight after 4–6 years, with a mean BMI of 30.5 kg/m² (range: 24.6–34.5 kg/m²) and a mean BMI-SDS of +2.1 (range: 0.7–2.9). All data are summarized in Table 2.

The diet was well tolerated. Hematologic and chemistry data were normal before starting the diet and remained normal over the 5-year diet period. No psychological symptoms or mood changes occurred during the study period. School performance was not impaired by the diet.

We did not observe any coronary events in our adolescent PWS patients. Other cardiovascular risk factors, such as hypertension, hypercholesterolemia, type 2 diabetes, and smoking, were not present in these patients.

**TABLE 2**
**Anthropometric Data***

| | Patient | Age (years) | Height-SDS | BMI (kg/m²) | BMI-SDS | Weight-for-Height Index (%) |
|---|---|---|---|---|---|---|
| At start of diet | 1 | 14.1 | –1 | 41.5 | 3.7 | 243 |
| | 2 | 15.5 | –1.5 | 36.8 | 3.4 | 207 |
| | 3 | 16.6 | –3.3 | 40.2 | 3.6 | 236 |
| | 4 | 18.9 | –3.5 | 55.5 | 4.5 | 339 |
| | 5 | 14.9 | –2.6 | 32.4 | 2.8 | 190 |
| | Mean | 16 | –2.4 | 41.3 | 3.6 | 243 |
| After ~1 year | 1 | 15.5 | –1.8 | 38.2 | 3.5 | 218 |
| | 2 | 17.2 | –1.9 | 30.2 | 2.5 | 166 |
| | 3 | 17.6 | –3.4 | 29.7 | 2.4 | 174 |
| | 4 | 20 | –3.5 | 41 | 3.6 | 250 |
| | 5 | 16.4 | –2.5 | 31.2 | 2.6 | 181 |
| | Mean | 17.3 | –2.6 | 34.1* | 2.9* | 197.8* |
| After ~2 years | 1 | 16.3 | –2.2 | 37.9 | 3.4 | 214 |
| | 2 | 17.5 | –1.8 | 30 | 2.4 | 164 |
| | 3 | 18.3 | –3.5 | 26.7 | 1.7 | 157 |
| | 4 | 20.6 | –3.5 | 38 | 3.3 | 232 |
| | 5 | 17.4 | –2.4 | 32.6 | 2.7 | 188 |
| | Mean | 18 | –2.7 | 33* | 2.7* | 191* |
| After 4–6 years | 1 | 19.1 | –2.4 | 34.5 | 2.9 | 193 |
| | 2 | 21.8 | –1.7 | 24.6 | 0.7 | 133 |
| | 3 | | | Not available | | |
| | 4 | | | Not available | | |
| | 5 | 19.2 | –2.3 | 32.5 | 2.6 | 187 |
| | Mean | 20 | –2.1 | 30.5* | 2.1* | 171* |

* $p < 0.01$ (compared to start of diet).

## DISCUSSION

PWS is one of the most common genetic causes of morbid obesity and is associated with a high incidence of sudden death, suspected to be cardiopulmonary in origin. So far, pharmacologic intervention with appetite suppressants (sibutramine) or antiabsorptive agents (orlistat), or with topiramate, are ineffective in patients with PWS[10]. To our knowledge, this is the first medium-term follow-up report about an effective dietary intervention in obese adolescents with PWS. Even after a short period of dietary treatment, a positive effect on BMI-SDS was observed.

Normal body proportion in PWS adolescents is a good predisposition for more physical activity, another important factor for weight control. In general, tailored exercise programs three to five times weekly are recommended for patients with PWS.

Secondarily, the success of the diet may be based on the close supervision of the patients in a foster home. Therefore, “food intake monitoring” plays another key role in the dietary intervention of PWS patients.

Meals containing large amounts of carbohydrates (especially mono- and disaccharides) can quickly raise blood sugar levels (meals with a high glycemic index) and are known to increase appetite. Meals with large amounts of fat slow down stomach emptying into the intestine, resulting in retarded absorption of carbohydrates with relatively low blood sugar levels. In PWS, delayed gastric emptying may relate to gastroparesis due to ineffective stomach contractions. Therefore, we believe that our macronutrient diet with 25% proteins, 20% fat, and 55% modified carbohydrates was decisive in controlling hyperphagia and food-craving behavior in PWS adolescents. Historically, weight maintenance in children with PWS has been reported as an intake of 8–11 kcal/cm of height, whereas normal children require 11–14 kcal/cm/day for adequate growth[4,11]. The overall goal of nutritional management of PWS is to provide optimal nutrition for health and growth, and to control weight at the same time. A dietary intervention also has to account for the changed body composition of PWS patients with increased fat mass and decreased lean body mass, muscle mass, and bone density.

In conclusion, energy expenditure and obesity can be controlled in patients with PWS, but a lifetime commitment and close monitoring are required.

Some characteristics of obesity in PWS patients are not common to severe obesity itself: total fat mass is increased, but visceral fat deposits are reduced[12]. Obesity, diabetes, and hypogonadism are well-established risk factors for cardiovascular disease. It is well recognized that PWS patients are at risk for premature death due to obesity-related respiratory failure and pulmonary hypertension. Obstructive sleep apnea, hypertension, and type 2 diabetes are common in adult PWS patients. In a recent study by Patel et al.[13], significantly raised CRP levels and cardiac microcirculatory dysfunction were found in adult PWS patients. Therefore, it is essential to achieve weight control in PWS patients.

## SUMMARY

Even in PWS adolescents who are already overweight, a well-supervised, special, strict, fat-reduced, and carbohydrate-modified diet, with 10 kcal/cm height, is effective in reducing weight over a 2- to 4-year period.
